# The Genomic Signature of Population Reconnection Following Isolation: From Theory to HIV

**DOI:** 10.1534/g3.115.024208

**Published:** 2015-11-04

**Authors:** Nicolas Alcala, Jeffrey D. Jensen, Amalio Telenti, Séverine Vuilleumier

**Affiliations:** * Department of Ecology and Evolution, University of Lausanne, Lausanne CH-1015, Switzerland; † Department of Biology, Stanford University, Stanford, California 94305-5020; ‡ School of Life Sciences, Ecole Polytechnique Fédérale de Lausanne, Lausanne CH-1015, Switzerland; § Genomic Medicine, The J. Craig Venter Institute, 4120 Capricorn Lane, La Jolla, California 92037; ** Institute of Microbiology, University Hospital and University of Lausanne, Lausanne CH-1011, Switzerland

**Keywords:** admixture, migration, coalescent, site frequency spectrum, HIV

## Abstract

Ease of worldwide travel provides increased opportunities for organisms not only to colonize new environments but also to encounter related but diverged populations. Such events of reconnection and secondary contact of previously isolated populations are widely observed at different time scales. For example, during the quaternary glaciation, sea water level fluctuations caused temporal isolation of populations, often to be followed by secondary contact. At shorter time scales, population isolation and reconnection of viruses are commonly observed, and such events are often associated with epidemics and pandemics. Here, using coalescent theory and simulations, we describe the temporal impact of population reconnection after isolation on nucleotide differences and the site frequency spectrum, as well as common summary statistics of DNA variation. We identify robust genomic signatures of population reconnection after isolation. We utilize our development to infer the recent evolutionary history of human immunodeficiency virus 1 (HIV-1) in Asia and South America, successfully retrieving the successive HIV subtype colonization events in these regions. Our analysis reveals that divergent HIV-1 subtype populations are currently admixing in these regions, suggesting that HIV-1 may be undergoing a process of homogenization, contrary to popular belief.

Past population demographic events leave distinctive signatures in DNA sequence polymorphisms and gene genealogies (Tajima 1989; Simonsen *et al.* 1995; Schneider and Excoffier 1999; Excoffier 2004; Zeng *et al.* 2006; Gutenkunst *et al.* 2009; Naduvilezhath *et al.* 2011). For example, a population bottleneck increases the variance of coalescence times and reduces the number of variants at low frequency in the site frequency spectrum (SFS) (Tajima 1989). Population expansion creates star-shaped genealogies and increases the mean number of variants at low frequency in the SFS (Tajima 1989; Slatkin 1996). Population sub-division leads to long internal branches in the genealogy and increases the number of fixed variants (Harpending *et al.* 1998). It also results in “structured” genealogies in which the means and variances of coalescence times (*e.g.*, time to the most recent common ancestor) are large, resulting in an excess of intermediate frequency variants in the SFS (Wakeley 1999). Balancing selection leads to a similar structure, and to an excess of variants at intermediate frequency (Tajima 1989; Fay and Wu 2000; Barton and Etheridge 2004; Zeng *et al.* 2006). Directional selection leads to genealogies with both star shapes (leading to the common ancestor of the selected lineages) and long branches (due to remaining ancestral lineages and/or recombination with the selected haplotype), and generates an excess of variants at both low and high frequency (Barton 1998).

Consequently, DNA sequence polymorphisms are increasingly used to infer past demographic events and have successfully reconstructed past histories of many organisms, including humans (Gravel *et al.* 2011; Reich *et al.* 2012). Some of the first methods proposed were developed to infer population growth rates (Slatkin and Hudson 1991) or for specific demographic scenarios (*e.g.*, Nielsen and Wakeley 2001; Hey 2010). Recently developed approaches allow for the inference of a wide range of demographic parameters (*e.g.*, Strimmer and Pybus 2001; Beaumont *et al.* 2002; Drummond *et al.* 2005; Gutenkunst *et al.* 2009; Excoffier *et al.* 2013). However, inferring reconnection of diverged populations after isolation remains difficult, as models of past isolation with recent contact are challenging to distinguish from models of ancient population separation with a continuous exchange of migrants. Also, parameter inference requires *a priori* knowledge of a population split (Hey 2010), and the time at which migration event(s) occurred has thus far been difficult to estimate (Sousa *et al.* 2011; Strasburg and Rieseberg 2011). Nielsen and Wakeley (2001) first proposed a method to infer time of divergence (IM model), the migration rate between the two populations, as well as the relative sizes of the populations. This approach was extended to account for the divergence of multiple populations (Hey 2010) and temporal reduction of gene flow following an initial population split (Wilkinson-Herbots 2012). Though others have discussed the expected signature of a reconnection event following a past isolation period (Becquet and Przeworski 2009; Sousa and Hey 2013), detailed theoretical formalization is still lacking. Besides this, the use of the aforementioned methods for general inference is also challenging as they are difficult to apply when the number of sampled populations is large (Beaumont *et al.* 2002; Drummond *et al.* 2005; Gutenkunst *et al.* 2009; Lohse *et al.* 2011; Excoffier *et al.* 2013), and when multiple sampled time-points are available (Excoffier *et al.* 2013; Foll *et al.* 2014). Further, they often rely on a specific evolutionary model, such as the Wright-Fisher model (*e.g.*, Gutenkunst *et al.* 2009; Lohse *et al.* 2011; Excoffier *et al.* 2013) which might not be representative of, for example, the skewed offspring distributions found for marine species or viruses (Tellier and Lemaire 2014)

Past population isolation and subsequent reconnection or secondary contact are common in natural populations and can have a drastic impact on species’ evolutionary histories. Anatomically, modern humans are thought to have admixed with Neanderthal populations after a period of isolation between the African and European continents (Green *et al.* 2010; Patterson *et al.* 2012; Sankararaman *et al.* 2012). Similarly, African and non-African populations of *Drosophila melanogaster* also experienced a period of isolation, followed by subsequent admixture (Pool *et al.* 2012). Domesticated species of both plants and animals are isolated from their wild relatives, and often experience subsequent genetic exchange (*e.g.*, in crops; Ellstrand *et al.* 1999). Importantly, isolation and subsequent reconnection events are also common features in viral histories, including the Influenza A virus, Human Immunodeficiency Virus (HIV) and Human Cytomegalovirus (HCMV; Renzette *et al.* 2013), where dependency on the host cell and other features of the life cycle isolate viral populations within hosts, groups of hosts, or host species in-between infections. For example, reassortment between avian and human influenza viruses caused the pandemic outbreaks in 1957 and 1968, and a reassortment of swine, avian and human influenza viruses was responsible for the 2009 pandemic (Hsieh *et al.* 2006; Garten *et al.* 2009; Flahault and Zylberman 2010).

As in influenza, events of HIV isolation and reconnection are common and can be observed at different spatial and temporal scales. Indeed, tracing the origin of HIV has confirmed that cross-species transmission of SIVs from other primates to humans, acting as sources of HIV, occurred several times over the past 100 years (Liégeois *et al.* 2014). The virus remained isolated for a very long period and adapted rapidly in humans, which ultimately rose to epidemic levels (Korber *et al.* 2001; Tebit and Arts 2011). Circulating HIV populations in humans are strongly divergent. Two major types of HIV exist (HIV-1 and HIV-2) and both have diversified into several groups or subtypes (Robertson *et al.* 2000). The HIV pandemic is mainly caused by HIV-1 Group M, which is composed by divergent (up to 35% sequence divergence) subtypes (named A–D, F–H, J, and K, Gaschen *et al.* 2002). The HIV epidemic is associated with the worldwide movements of people that allowed HIV-1 subtype colonization events (Tebit and Arts 2011; Hamelaar *et al.* 2011). In the course of their evolution in human populations, HIV-1 subtypes experienced several large-scale isolation events within both transmission risk groups (Su *et al.* 2000; Pang *et al.* 2012; Han *et al.* 2013; Li *et al.* 2013) and countries (Bello *et al.* 2011; Castro-Nallar *et al.* 2012). Subsequent reconnection then occurred through coinfection, superinfection, and recombination (Tebit and Arts 2011; Vuilleumier and Bonhoeffer 2015). Those reconnection events are the source of emergence of many new recombinant forms which constitute a major challenge for vaccine development and are at the origin of new epidemics (Tebit and Arts 2011; Feng *et al.* 2013). The multiple colonization events of HIV-1 that occurred in Asia and in South America are representative of this trend (Hamelaar *et al.* 2011). Indeed, with an estimated 4.8 million people living with HIV as of 2011, Asia is the second most HIV-affected region worldwide (Zhang *et al.* 2013). Historical epidemics in Asia are associated with transmission events to various risk groups; HIV-1 subtypes B and C were first detected in the mid-1980s and early 1990s, respectively, in the Injecting Drug Users (IDUs) risk group (Lu *et al.* 2008), and then spread to other populations. Subtype CRF01_AE was first detected in the Men who have Sex with Men (MSM) risk group in the 1990s, then spread to other groups and became the most prevalent HIV-1 subtype in many parts of China. Similarly, the epidemic clade circulating in South America is derived from subtype B viruses that migrated out of Haiti around 1969 (1966–1972) and spread through the world (Bello *et al.* 2011). Later, a single-founder subtype F1 strain, introduced in Brazil, spread and recombined with local subtype B viruses to form the HIV-1 BF1 and F1 epidemics (Aulicino *et al.* 2007).

The present study identifies specific temporal signatures of reconnection of diverged populations in two broadly used summaries of DNA polymorphism: the distribution of pairwise nucleotide differences (via coalescent theory), and the Site Frequency Spectrum (SFS, via simulation). We also describe how past isolation can influence commonly used SFS-based test statistics (Tajima’s *D*, Fu and Li’s $D^{\text{*}}$ and $F^{\text{*}}$, Fay and Wu’s *H*, Zeng *et al.*’s *E*), and discuss the specificity and the robustness of the signature relative to other demographic and selective processes. Then, using theoretical results and full genome polymorphism data, we reconstruct the successive invasions of HIV-1 subtypes in China and in South America and analyze the subsequent dynamics of genome composition. We find extensive admixture between HIV-1 subtypes, potentially leading in the long term to homogenization of HIV-1 genomes in these regions.

## Materials and Methods

Using coalescent theory, we first present methods for detecting the signature of population reconnection after isolation on the distribution of pairwise nucleotide differences. Then, in the second section, we use coalescent simulations to derive the signature of population reconnection after isolation on the SFS. The third section highlights the robustness of these signatures to alternative demographic scenarios. Finally, the fourth section presents a data application for these methods: inference of recent HIV-1 evolution in China and in South America.

### Theoretical signature

In our model, we consider *d* previously connected populations that became isolated at time $T_{iso}$ and then reconnected at time $T_{reco}$. The scaled migration rate *M* between populations (*i.e.*, twice the number of migrant genes per population per generation) is the same before and after the isolation period and mutations follow a Poisson process of rate θ/2 (Kimura 1969). We used an infinite sites model for the analytical investigations, and for the simulations we considered a finite sites model with a number of sites corresponding to HIV-1 genome size (9719bp, from the reference HXB1 sequence). Our developments are based on the Wright-Fisher model (Fisher 1930; Wright 1931), where each population has a constant size *N*. For analytical simplicity, we considered that times $T_{iso}$ and $T_{reco}$ are scaled in units of *N* generations, so $T_{iso}=1$ corresponds to an isolation event that occurred *N* generations ago. We derived an exact theoretical formula for the distribution of pairwise nucleotide differences in nonrecombining genomic regions (*Appendix A*), and simulated this distribution using coalescent simulations (software ms; Hudson 2002) in the case of recombining regions. We computed the power to sample sequences from each mode of the distribution using equations A.6a–A.6b (Supporting Information, Figure A in File S1).

We generated the SFS through time following isolation and reconnection events using coalescent simulations. We studied the *total SFS* (considering pooled samples from all populations); in the main text, we present results for equal sample sizes in each populations, and we present results for alternative sampling schemes (Figures B and C in File S1). We also studied the joint SFS between pairs of populations. We assessed the temporal changes of the total SFS using the visual test of Nawa and Tajima (2008), which is based on the difference between the expected SFS under neutrality in a population with constant size, and the observed SFS. For each frequency i/n, where *n* is the sample size, the transformation represents ${\hat{\theta }}_{i}=i\xi_{i}$, instead of the corresponding number of variants $\xi_{i}$. This simple test enables one to easily detect which frequencies present an excess or a deficit of variants. We also derived an optimal test statistic to detect the signature of population reconnection from the SFS (Text A and Figure D in File S1) and compute the power of common neutrality tests to detect such events (Figure E in File S1).

### Robustness analysis of the theoretical signature

We then analyzed the sensitivity of our results to departures from the infinite sites model (Figure F in File S1), from the constant population size model (Figure G in File S1) and from the Wright-Fisher model (Figure H in File S1). To account for repeated bottlenecks (*e.g.*, during transmission events), extinction-recolonization dynamics, and rapid adaptation, we simulated the distribution of pairwise nucleotide differences under a scenario of reconnection of isolated populations under a beta-coalescent model. Beta-coalescent models have recently been proposed to model the genealogies of many organisms with a large variance in the number of offspring per individual (Birkner and Blath 2008; Tellier and Lemaire 2014), in particular viruses (Neher and Hallatschek 2013). We extended the algorithm from Birkner and Blath (Birkner and Blath 2008) – which generates samples under a beta-coalescent at equilibrium – to include an island model of migration (each lineage migrates to another population at rate *M*) and an isolation period. We assumed that coalescence processes follow the beta-coalescent at all times: before isolation, during isolation and after reconnection. In addition to mutation and migration rates, beta-coalescents rely on a parameter α (0≤α≤2) that gives the probability of multiple lineages coalescing at the same time. We present the results for four different parameter values: α=0 (equivalent to the classic Kingman coalescent), α=0.5, α=1 [corresponding to the Bolthausen-Sznitman coalescent, used to model strong positive selection (*e.g.*, Neher and Hallatschek 2013)], and α=1.5.

We also compared the signature of population reconnection after isolation with that of a closely related model: the Isolation with Migration model [IM (*e.g.*, Takahata 1995; Wakeley 1996; Rosenberg and Feldman 2002)], using coalescent simulations (Figure I in File S1).

### HIV data analysis

We used 1646 whole genome HIV-1 samples from subtypes B, C, F1 and CRF01_AE and recombinant forms between these subtypes (Table A in File S1). We used the subset of genomes from China sequenced between 2005 and 2009 (297 sequences; Table B in File S1). For the study in South America (Brazil and Argentina), we used data available between 1999 and 2005 (128 sequences; Table C in File S1). Finally, for the worldwide PCA analysis, we used all 1646 genomes available.

For the Chinese and South American data, we identified HIV-1 populations (subtype clusters) by Discriminant Analysis of Principal Components (DAPC) (Jombart *et al.* 2010). Our investigations considered dominant subtypes B, C, CRF01_AE and F1 and their associated recombinants. Each cluster encompassed a dominant subtype and some recombinant forms between subtypes. We found strong support for four groups using the Bayesian Information Criterion (ranging from 2 to 100 PCA axes) that are independent of sampling year (See Figure J in File S1 for a neighbor joining tree of the sequences where the different clusters and sampling years are represented). We then computed the distribution of pairwise nucleotide differences and the total and joint SFS for the major circulating HIV-1 subtypes in China and South America.

### Computing the temporal patterns of genetic variation of HIV subtypes

We computed the distribution of pairwise nucleotide differences, and the total and joint SFS for the major circulating HIV-1 subtypes in China and South America. The distribution of pairwise nucleotide differences within (within-population pairwise differences) and between clusters (between-population pairwise differences) were generated for each nonrecombinant block between subtypes that we identified with *jpHMM* (Schultz *et al.* 2009). In total, 19 and 24 blocks were identified in sequences sampled in China and South America, respectively; the 5%, median and 95% quantiles of block length were 111bp, 220bp and 1333bp, respectively. Detection of bimodality in the distribution, the signature of a past isolation event, was performed by fitting a Gaussian Mixture Model using the Expectation-Maximization algorithm from Meng and Rubin (1993) as implemented in the R package *mixtools* (Benaglia *et al.* 2009). Gaussian distributions are used to estimate positions of the mode as distributions are symmetric (the 5% and 95% quantiles of the skewness of all components are -1.05 and 1.05, respectively). The algorithm also provides posterior probabilities of membership into modes, which is used to identify genomes that have bimodal distribution (*i.e.*, indicating admixture). We analyzed the signatures in the pairwise nucleotide differences by comparing HIV genome signatures with the expected results, assuming either small linked sequences or large recombining sequences.

The total and joint SFS were computed using subtype J as an outgroup. Results were robust to the chosen outgroup (Figure K in File S1). Note that the distinction between recombining and nonrecombining sequences does not apply for the SFS, as the expectation (and the maximum likelihood estimate) of the SFS is not affected by recombination: only its variance is affected (Gutenkunst *et al.* 2009). We tested the significance of the temporal changes of the SFS using a bootstrap test (Figure L in File S1).

### Data availability

All data used are from the Los Alamos HIV database (http://www.hiv.lanl.gov/) (Table A in File S1).

## Results

### Bimodality in pairwise nucleotide differences as a signal of population reconnection after isolation

**Figure 1:**
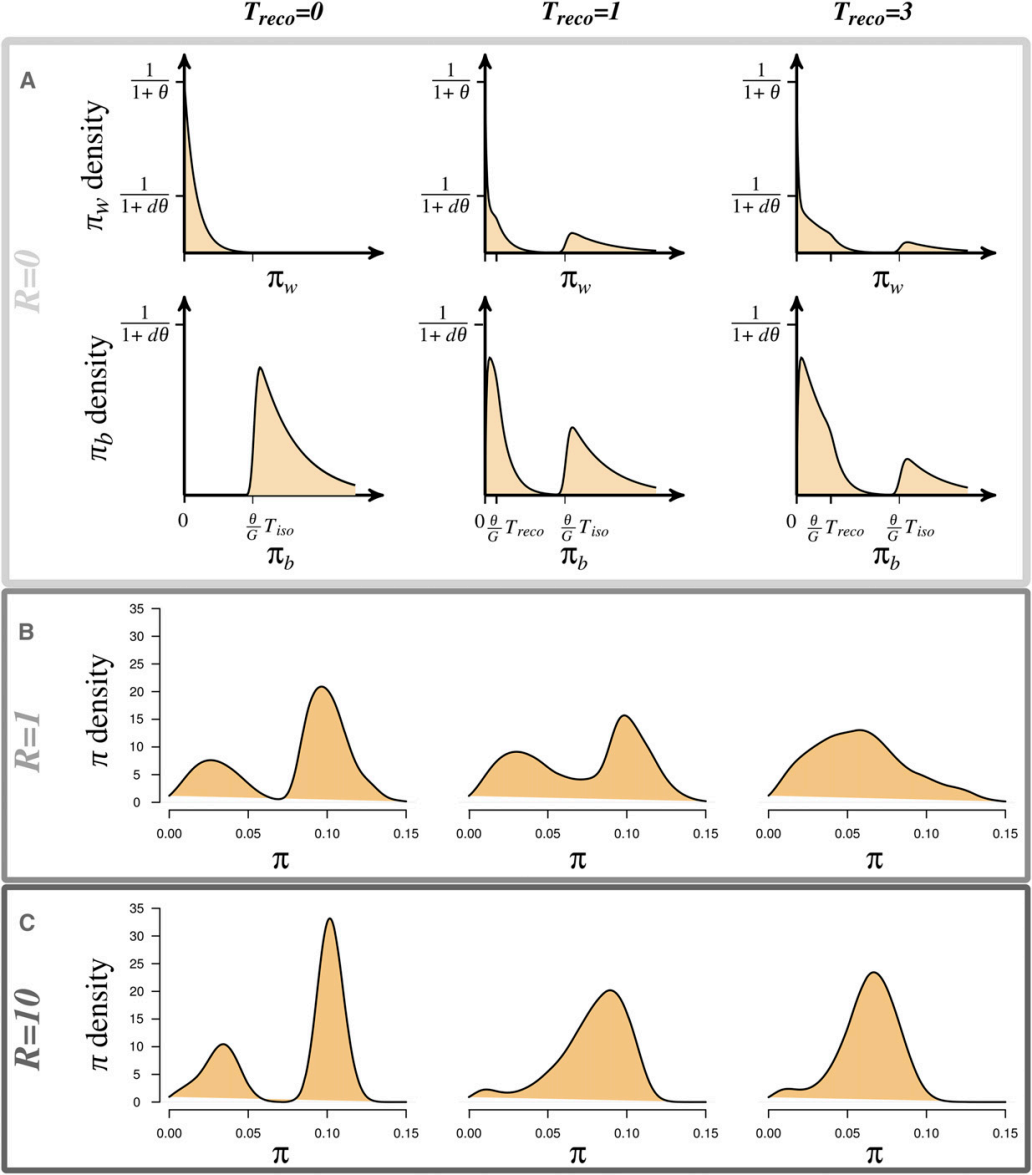
Signature of reconnection of previously isolated populations on the distribution of pairwise nucleotide differences as a function of time since the reconnection event $T_{reco}$ (columns), (A) within-$\pi_{w}$ and between-population $\pi_{b}$, (B–C) total π pairwise nucleotide differences. In panel A, we consider a sequence of size *G*, with *G* larger than the number of segregating sites without recombination; plots in panel A are computed using equation A.5. In panels B and C, we consider a sequence of 9719 bp (length of the reference sequence for HIV-1, HXB1) with various mean numbers of recombination events per generation, R=1 and R=10. Nucleotide differences are considered from a sample of 16 sequences and each plot represents the mean distribution over 2000 replicate simulations. Parameters are d=3 populations, duration of the isolation period $T_{iso}=6$, scaled migration rate M=5, scaled mutation rate θ=100.

Two distinct signatures of population reconnection after isolation were found in the temporal distribution of pairwise nucleotide differences: bimodality and increased variance of the first mode (Figure 1). Bimodality was observed in these distributions (both within and between populations, $\pi_{w}$ and $\pi_{b}$) only when a population reconnection occurred (Figure 1A). This bimodality reflects the different possible origins of the two sequences sampled: the two sequences either have a recent common ancestor (*i.e.*, after the reconnection event; first mode in Figure 1A) or an ancient common ancestor (before the isolation event; second mode in Figure 1A). Bimodality was also observed in the distributions of total pairwise nucleotide difference (considering all populations, π) after a reconnection event but, in this case, bimodality was also observed when populations were completely isolated ($T_{reco}=0$, Figure 1, B and C). To differentiate a connection from an isolation event, bimodality needs to be associated with a temporal increase of the variance of the modes in the distribution (which depends on the recombination rate; see Figure 1). Although testing for increase in variance requires data from different time-points, it has the advantage of not requiring *a priori* knowledge of the origin of the samples (*i.e.*, being within or between populations).

Population reconnection after isolation can thus be detected by testing for bimodality and a temporal increase of the variance of the modes. Power to detect bimodality, with high probability (95%) (Figure A in File S1), depends on the sample size, the duration of the reconnection period, and the amount of gene flow among populations (scaled migration rate). As shown in Figure A in File S1, it is easier to detect a reconnection event from the distribution of pairwise nucleotide differences between populations than within populations. Indeed, the second mode in the distribution of pairwise nucleotide differences was larger between population ($\pi_{b}$) than that of within population ($\pi_{w}$, Figure 1A). This difference in mode size increases when migration is weak or intermediate (M≤1) and the reconnection event is not recent (Figure A in File S1). Ancient reconnection events can be detected even with small sample sizes (n≃10) when migration is moderate or strong (M≥1; Figure A in File S1). To evaluate the temporal increases in variance of the mode, no further development is required as the Bartlett test (Bartlett 1937) can be used.

Thus, we show that distinctive signatures of past isolation and reconnection events can be identified on the distribution of the number of nucleotide differences between pairs of genomes sampled (within, between, or in the total population). We also determine the parameter ranges for which this signature can be detected (sample size, migration rate, and time since reconnection).

### The signature of population reconnection after isolation on the Site Frequency Spectrum (SFS)

**Figure 2:**
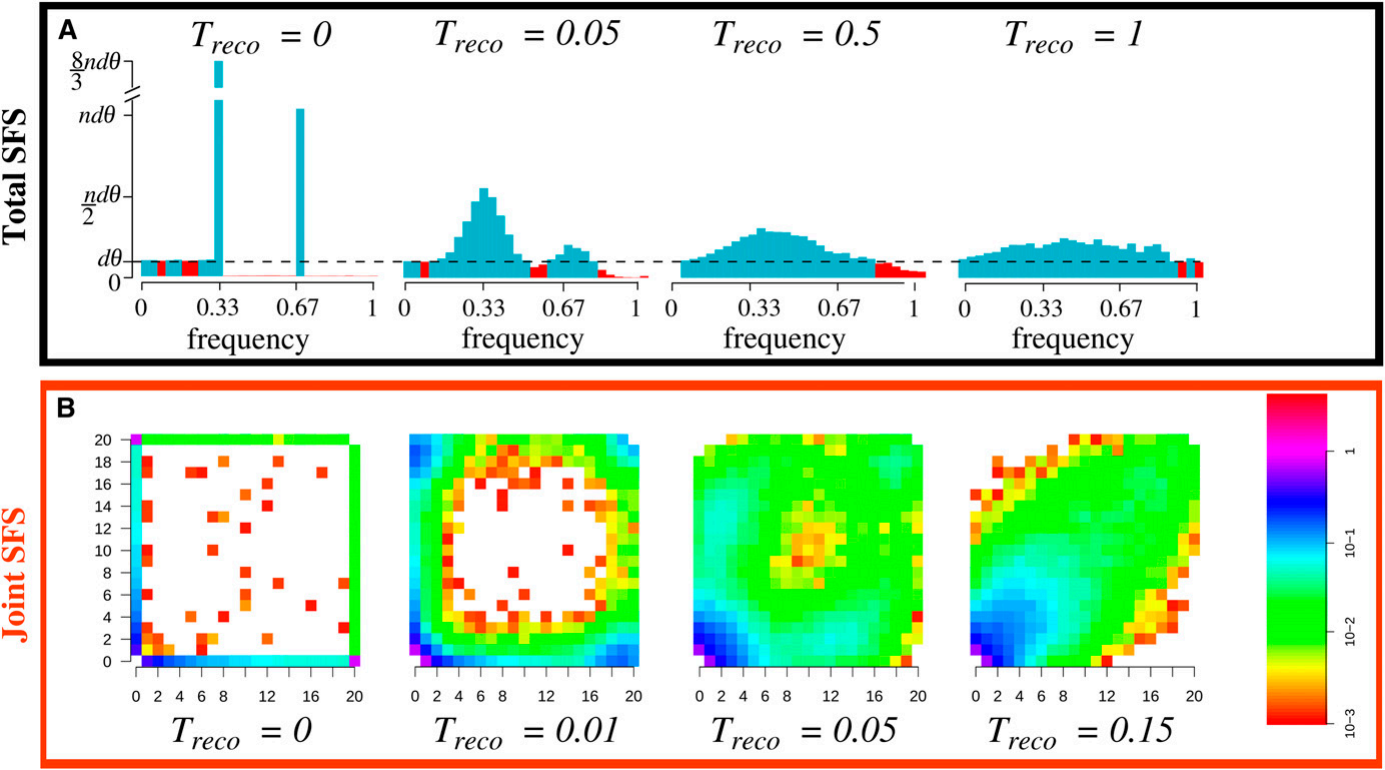
Signature of a reconnection event between previously isolated populations on (A) the total and (B) joint site frequency spectrum (SFS), as a function of the number of generations since the reconnection event, $T_{reco}$. (A) The SFS representation of Nawa and Tajima (2008) is used; the expected value of a single panmictic population at equilibrium is given by a straight line (dashed line), and deviations from a straight line indicate an excess (in blue) or deficit (in red) of variants at a given frequency. Parameters are d=3 populations, n=16 sampled genes per population (see Figures B and C in File S1 for alternative sampling schemes), with θ=100. Means are over 2000 replicates.

When a population is at mutation-drift equilibrium, the distribution of its alleles, or SFS, has a characteristic shape (dotted line Figure 2A). When populations experience demographic changes, departures from this characteristic shape are expected and the size and the position of those changes are informative as to the demographic change. Consistent with the expectation, we found a large excess of variants at intermediate frequencies on the SFS following reconnection events between previously isolated populations (Figure 2). This excess was due to exchanges of variants among populations that were once fixed in one or several populations during isolation. The size and the position of this excess is indicative of the number of populations that have reconnected and of the timing of the reconnection event (Figure 2A), and can be identified by the visual test of equilibrium neutrality proposed by Nawa and Tajima (2008). We predict that in the total SFS (SFS obtained with merged samples from all populations), shortly after a reconnection event one would expect narrow, high peaks of specific variants. These peaks are found at frequencies i/d, where *d* is the number of sampled populations and 1≤i<d, when populations have equal sample sizes. In Figure 2A, peaks can be seen at frequencies 1/d and 2/d (d=3). With unbalanced sample sizes, the number of observed peaks can be higher (up to $2^{d}-2$ peaks; see Figure B in File S1).

The temporal patterns in the joint SFS (Figure 2B) are also strongly informative as to the occurrence of reconnection events and as to the amount of gene flow among populations since the reconnection event. When populations are isolated, each has its own segregating variants. This translates, in the joint SFS, into variants being restricted to the axes of the joint SFS [classes (20,*i*), (*i*,20), with *i* ranging from 1 to 20 in Figure 2B]. Then, following the reconnection event, gene flow distributes the variants among populations to the point that they occur at similar frequencies in each population and the genetic compositions of populations are fully homogenized. Consequently, in the joint SFS, several variants move progressively away from the axes toward the diagonal of the joint SFS, as expected in a structured population at equilibrium (last column Figure 2B).

To robustly detect the excess of variants on the SFS generated by a reconnection event, we derived an optimal test statistic, denoted $T_{\Omega }$, using developments provided by Ferretti *et al.* (2010) and Achaz (2009) (File S1). Although use of this test is restricted to recent reconnection events that follow long periods of isolation, it performs better than commonly used statistics such as Tajima’s *D* (Tajima 1989), Fu and Li’s $D^{\text{*}}$ and $F^{\text{*}}$ (Fu and Li 1993), Fay and Wu’s *H* (Fay and Wu 2000), Zeng *et al.*’s *E* (Zeng *et al.* 2006). Indeed, even with long isolation periods, those statistics have less than 60% power to detect reconnection events (Figure E in File S1). The results presented in this section apply to both recombining and nonrecombining sequences, as the expected SFS is the same in both cases (Gutenkunst *et al.* 2009). Also, the power estimates from the tests based on nonrecombining sequences are conservative as they correspond to the case with the highest variance (Achaz 2009) and thus the lowest power.

Thus, we show that distinctive signatures of past isolation and reconnection events can be identified in the SFS as well as in the joint SFS. Namely, the SFS has an excess of variants, the size and position of which are indicative of the number of previously isolated populations and of the timing since the reconnection event. The temporal dynamic of the joint SFS is informative as to the level of gene flow between populations.

### Robustness of identified signatures to alternative demographic scenarios

The signatures of population reconnection identified here, *i.e.*, bimodality and temporal increase of variance of peaks in the SFS, are robust to population expansion (Figure G in File S1), alternative evolutionary and population dynamic models such as repeated bottlenecks, rapid extinction-recolonization dynamics, recurrent positive selection or highly skewed offspring distributions (Figure H in File S1). Moreover, the signature is distinct from the signature of a closely related model: the Isolation with Migration model (IM) (Figure I in File S1).

The observed signatures are robust even when a massive population expansion occurs concomitantly with the reconnection event (Figure G in File S1). Such events modify the expected signature only by presenting an excess of low frequency variants compared to what is expected with populations of constant size (as expected from the results of Tajima (Tajima 1989) in a single panmictic population). Replacing the classic Kingman coalescent model (Wright-Fisher model) with a beta-coalescent model does not alter our results for values of the model parameter α (Figure H in File S1). Beta-coalescent models account for large variance in the number of offspring per individual, as expected under repeated bottlenecks, fast extinction-recolonization dynamics, or recurrent beneficial fixations (Neher and Hallatschek 2013; Tellier and Lemaire 2014). Such variance leads to an excess of low frequency variants and smaller peaks in the total SFS compared to Kingman’s coalescent model; this effect is larger for small α values (light-gray lines Figure H in File S1). However, having an excess of variants at low frequency does not alter the identified signature of reconnection on the total SFS (dynamics of the peaks). Also the temporal dynamics of the signature distinguish this pattern from that of an IM model. Indeed under the IM model, the size of the peaks in the SFS increases with time (Figure I.B in File S1), whereas the signature of a reconnection of isolated populations is characterized by a decrease in peak size with time (Figure 2A).

Thus, we show that, even though the signature at specific time-points may be confounded with that of other selective or demographic processes, the temporal signature of past isolation and reconnection identified is unlikely to be confounded, and provides a robust signal.

### Detection of past isolation and current reconnection of HIV-1 subtypes

We applied the conceptual and theoretical approaches via an analysis of the colonization history of different HIV-1 subtypes in Asia and in South America. Using data from the Los Alamos HIV database, we detected a strong signal of reconnection events between previously isolated HIV-1 subtypes in these populations.

**Figure 3:**
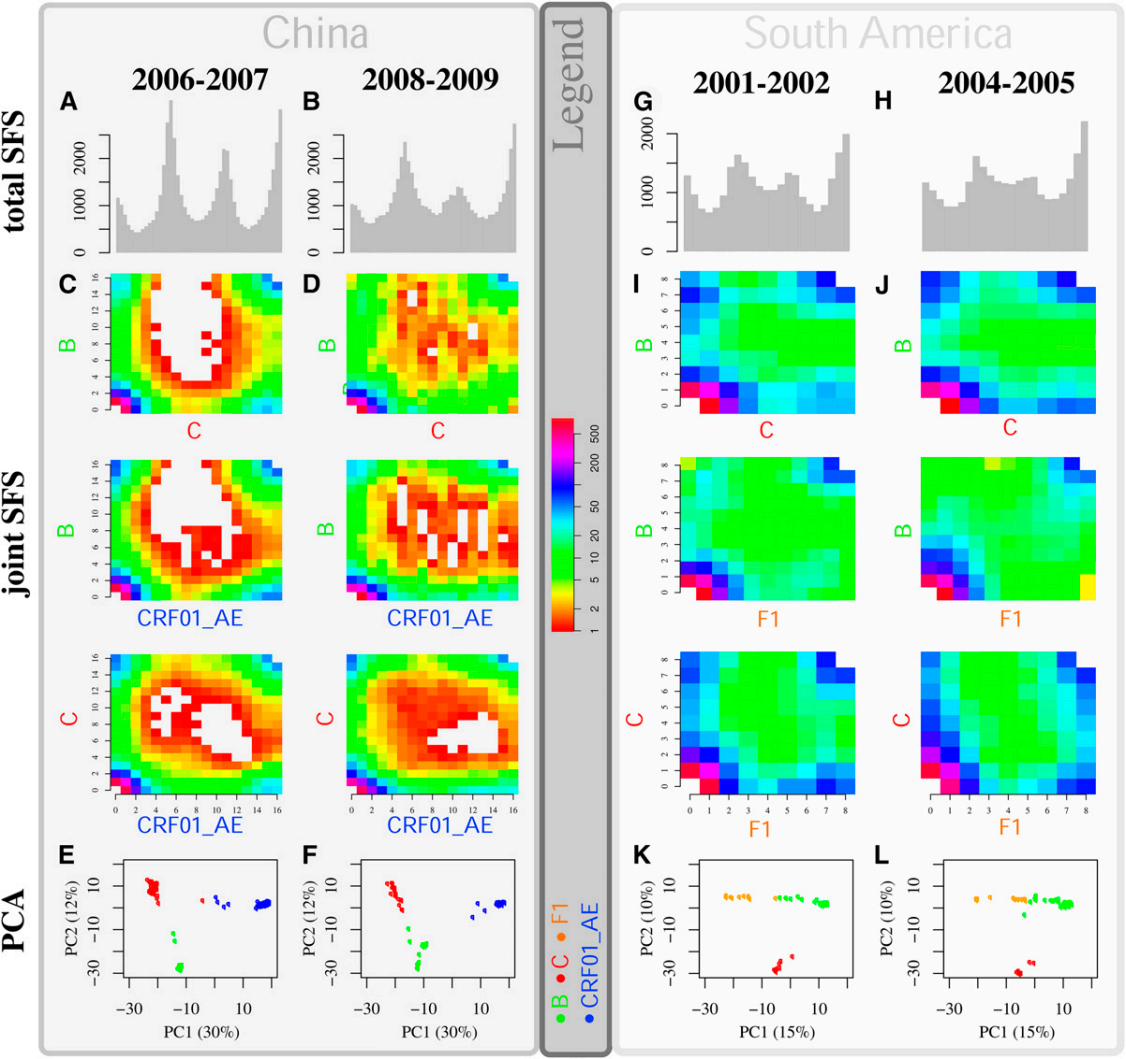
Signatures of HIV-1 subtype reconnection events on the Site Frequency Spectrum (SFS). Results are presented for China (left major panel) and for South America (right major panel). Two time points of HIV-1 sequences sampled are considered in China (Table B in File S1) and in South America (Table C in File S1) (see method section ‘HIV-1 genome analysis’). The total (pooled) SFS is presented in the first row following representation of Nawa and Tajima (2008), the joint SFSs of each pairwise group of HIV subtypes are presented in the second, third, and fourth row and, in the fifth row, the Principal Component Analyses (PCAs) are presented. Subtype J is used as an outgroup (see Figure K in File S1, results for alternative outgroups). Original subtypes (subtypes found early in the epidemics in Africa; at the corners) are used to define a triangle in the PCA space. The position of the HIV-1 genomes on the straight edges of the triangle suggests strong admixture between subtypes. For the total and joint SFS, we consider the mean value of 500 random samples of sequences in each subtypes cluster (see Figure J in File S1 for the different clusters). A sample of 16 sequences per subtype cluster is considered in China, and of eight sequences per subtype cluster in South America. The significance of the trends in the dynamics is tested using the optimal test statistic $T_{\Omega }$ derived in the SI (Figure L in File S1).

Past isolation between HIV-1 subtypes in China and South America is signaled by the excess of variants, with peaks at intermediate frequencies in the total SFS (Figure 3, A and B and G and H). These peaks reflect the presence of variants from the sampled population that diverged during isolation and were present at high frequencies in each previously isolated subtype population identified with cluster analysis (see *Results*).

**Figure 4:**
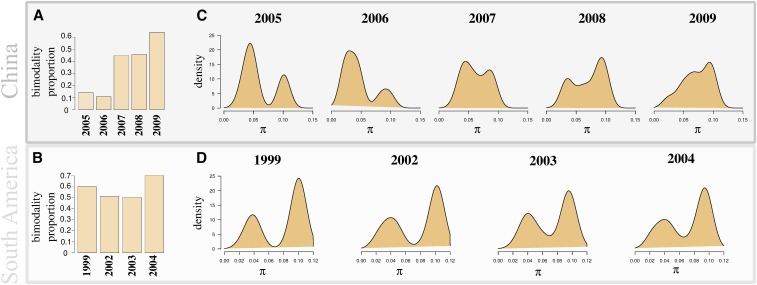
(A–B) Proportion of bimodality in pairwise nucleotide differences (between populations) detected in the genomes and (C–D) distribution of total pairwise nucleotide differences (all populations) of HIV-1 sequences for different time points in China (see Table B in File S1) and South America (see Table B in File S1). As expected under genomic admixture (Figure 1), the proportion of bimodality detected in the pairwise nucleotide differences between populations in HIV-1 genomes increased, and the variance of modes significantly increased with time in China (p<0.01, two-sided Bartlett test). We also see a (nonsignificant) trend of temporal changes in South America.

The reconnection of HIV-1 subtype populations in China and South America is indicated by the bimodality in the distribution of pairwise nucleotide differences between-populations (considering sequences of small length Figure 4). We found that a large proportion of the genome of HIV-1 displays bimodality (Figure 4). In China, this proportion was initially low but rapidly increased through time. Between 2006 and 2007, the proportion of bimodality increased fourfold (Figure 4), and increased a further 40% between 2008 and 2009. This suggests that the time series analyzed here captures a burst of HIV-1 subtype admixture. The dynamics of genomic admixture between HIV-1 subtypes in China is also highlighted by the temporal changes in the distribution of pairwise nucleotide differences and the SFS. The peaks formed by the excess variants (resulting from population admixture in the total SFS) decreased in size, and the values of optimal test statistics $T_{\Omega }$ significantly decreased (Figure L in File S1). We find a significant increase in the variance of the first mode in the distribution of pairwise nucleotide differences between 2005 and 2009 (p<0.01 two-sided Bartlett test). The joint SFSs for the two time points also indicate that the reconnection event and genome admixture is either recent or that genome admixture is limited but ongoing. Indeed, we can observe the excess of variants along the axes of the joint SFS shifting to the diagonal (Figure 3, C and D). In South America, the proportion of bimodality in HIV-1 samples from 1999 is large (60%) and remains approximately constant from 1999 to 2004, signaling a prior reconnection event. Patterns observed in the joint SFS suggest that HIV-1 subtype genomes are well homogenized overall; further, this process of homogenization seems to have either slowed or ceased. Indeed, no increase of variance in the modes of the distribution of pairwise nucleotide differences was detected (two-sided Bartlett test), and the total SFS shows only small changes between 2001 and 2005, with nonsignificant changes in values of optimal test statistics $T_{\Omega }$ (Figure L in File S1).

Genome admixture in China and in South America is also visible when the sampled genomes are projected together in a PCA space defined by original subtypes *i.e.*, which originate from the beginning of the HIV epidemic in Africa (Figure 3, E and F and K and L). Several HIV-1 genomes are present on the straight edges of the triangles defined by the ones at the origin. Temporal PCA patterns also suggest homogenization between subtypes B and C in China and between B and F1 in South America, as they move away from the pure subtypes in PCA space.

**Figure 5:**
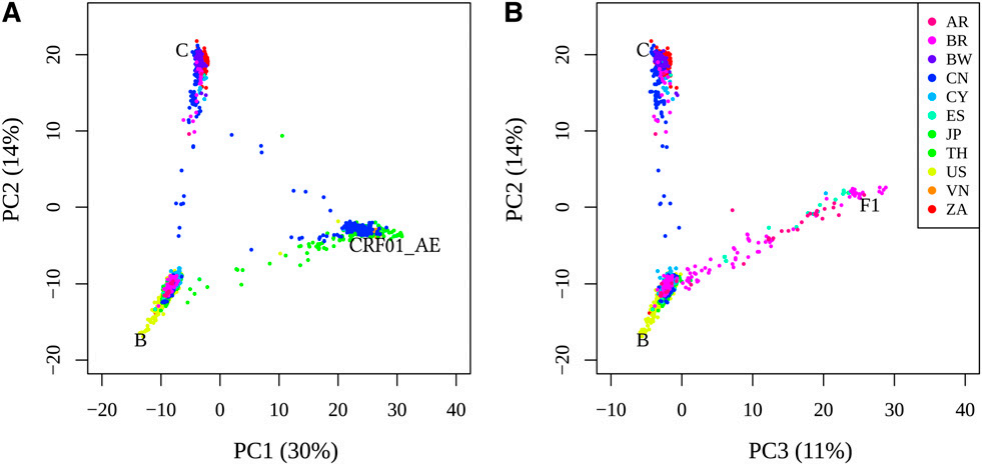
Projection of worldwide distributed HIV-1 sequences from subtypes B, C, CRF01_AE and F1, and URF and CRF recombinant forms between these subtypes. 1646 genome sequences (see Table A in File S1). (A) The first two axes of the principal component analysis (PC1 and PC2), and (B) the second and third axes of the principal component analysis (PC2 and PC3) defined by pure subtypes B, C, CRF01_AE and F1 (see method section ‘HIV-1 genome analysis’). The percentage of variance explained by each PC is indicated between parentheses. The position of a HIV-1 genome sequence along the axes of the triangle formed by three pure subtypes reflect admixture (proportion) between the subtypes (Ma and Amos 2012). Standard country codes are used: Argentina (AR), Brazil (BR), Botswana (BW), China (CN), Cyprus (CY), Spain (ES), Japan (JP), Thailand (TH), USA (US), Vietnam (VN), South Africa (ZA). Misrepresented sequences [squared cosine of the PC plane with the observation <0.05(Abdi and Williams 2010)] were excluded.

To provide insights on the generality and global importance of these results, we performed a PCA analysis of worldwide genomes from HIV-1 subtypes B, C, F1, and CRF01_AE (http://www.hiv.lanl.gov). Again we projected them in the PCA with original subtypes B, C, F1, and CRF01_AE as references (Figure 5). Consistent with the observations for Chinese and South American samples, we observed that HIV-1 genomes are positioned on the straight edges of the triangle formed by original subtypes, positioned at the corners, forming a continuum along the axes rather than discrete clusters within. Very few genomes are localized outside the edges of the triangle defined by the 3 original subtypes. This pattern not only signals that most of the circulating HIV-1 genomes experience constant gene flow from other subtypes but also represents the current spatial distribution of HIV-1 populations. It reflects, for example, the extensive admixture between subtypes B and CRF01_AE in Brazil and China, between B and CRF01_AE in Thailand, and B and F1 in Brazil, Argentina, and Spain.

## Discussion

We present an analytical framework to understand the evolutionary histories of organisms that are characterized by periods of isolation. Our theoretical investigations identify specific genomic signatures of past population isolation. We show, for example, that the number, size, and dynamics of modes in the distributions of the pairwise nucleotide differences in populations are informative on the existence of past isolation. Our results extend previous work on the distribution of pairwise coalescence times and pairwise nucleotide differences under a model of population split with or without migration (IM model). We show, for example, that under the reconnection model, the distribution of pairwise nucleotide differences has a temporal evolution which is distinct from the one expected under an IM model (Takahata 1995; Wakeley 1996; Rosenberg and Feldman 2002): the distribution is first bimodal and then tends to become unimodal with time. We also found that in the Site Frequency Spectrum (SFS), the presence, the position and the value variants in excess are informative as to the numbers of previously isolated populations, and the joint SFS characterizes the amount of genetic overlap between populations. Signatures identified here are robust to the presence of recombination, extinction-colonization processes, population bottlenecks, and expansion as well as skewed offspring distributions. Our developments add to the current literature in the field as we demonstrate that population reconnection after isolation is not fully captured by commonly used test statistics based on SFS classes of variants at high and/or low frequencies such as *D*, *H*, and *E* (Ferretti *et al.* 2010). Taken together, this provides critical information on the evolution of the genomic composition of populations.

We apply our theory in order to analyze the genome signature of successive colonization of HIV-1 subtypes in Asia and South America. In both regions, we reconstruct the successive colonization of HIV-1 subtypes, and find that they are followed by strong HIV-1 subtype admixture. We find, in these regions, that HIV diversification is followed by a temporal homogenization of HIV-1 genome composition. Our analysis highlights a large degree of admixture between circulating HIV-1 subtype genomes in China and South America, and most probably worldwide. These results suggest that genomic exchanges may indeed be relatively strong, and that HIV-1 is currently in a process of homogenization subsequent to isolation and diversification. HIV genome homogenization may seem to contradict the idea that HIV is undergoing a diversification process (Hamelaar *et al.* 2011; Tebit and Arts 2011; Feng *et al.* 2013). However, this homogenization is consistent with observed diversity in the HIV-1 population and the increased number of new HIV recombinant forms described worldwide (Hamelaar *et al.* 2011; Tebit and Arts 2011; Feng *et al.* 2013). Indeed, when isolated and diverged populations reconnect, a transient excess of genetic diversity is predicted in the resulting population (Alcala *et al.* 2013; Alcala and Vuilleumier 2014) as all the genetic material that accumulated during the isolation phase is shared among populations. However, this excess of genetic diversity is transient and with time, the diversity decreases (Alcala *et al.* 2013). In HIV-1, such processes occur through multiple recombination events among subtypes through coinfection or superinfection and are the source of novel recombinant forms. In the long term, successive recombination events will ultimately lead to the homogenization of the genomic composition of HIV-1. Homogenization might be further enhanced by selective forces imposed by the immune response or treatment acting on the HIV genome (Fellay *et al.* 2007; Pelak *et al.* 2011; Chikata *et al.* 2014). As many world regions are currently being newly colonized by different HIV-1 subtypes, it is expected that new recombinants may still emerge and HIV diversity will further increase. However, as demonstrated here, this is expected to be a transient state toward global homogenization.

We find signatures of reconnection events among populations of HIV-1 subtypes that were previously isolated both in China and South America. The signatures on HIV-1 DNA polymorphism suggest reconnection events of three previously isolated HIV-1 populations in both regions. These results align with the known epidemiologic history of HIV-1 in the two regions (Su *et al.* 2000; Yang *et al.* 2002; Aulicino *et al.* 2007; Lu *et al.* 2008; Takebe *et al.* 2010; Bello *et al.* 2011; An *et al.* 2012; Pang *et al.* 2012; Han *et al.* 2013; Feng *et al.* 2013) and are supported by recently identified recombinants in China (Feng *et al.* 2014; Wang *et al.* 2014; Wei *et al.* 2014; Hsi *et al.* 2014). To confirm this, we identified the genome sequences that signal the reconnection event. As expected, they reflect first the HIV outbreak in China in 1989: colonization of B and C subtypes (variance of the first peak in the SFS is generated by B and C subtypes). The subsequent colonization of CRF01_AE at the origin of the HIV outbreak in the late 1990s is also signaled in the SFS (a high proportion of CRF01_AE in the highest mode). We obtained similar concordance for the origin of the sequences forming the peaks and the successive subtypes’ colonization events in South America.

Our methods also appear robust to high mutation rates and the presence of selection. Indeed, we detect past HIV-1 history despite its extraordinary defining features: HIV has a small genome (about 9.8 kb in length), a short generation time (24 hr; Mohammadi *et al.* 2013), and a high mutation rate (about 0.002 substitutions/site/year; Korber *et al.* 2000; Schlub *et al.* 2014). It is also under strong selective pressure from the immune system (*e.g.*, Snoeck *et al.* 2011) and undergoes frequent bottlenecks followed by population expansion [during transmission (*e.g.*, Keele *et al.* 2008)]. Nevertheless, the signature observed here in the distributions of pairwise nucleotide difference and on SFS only slightly deviates from theoretical expectations. The robustness of these results may have a simple explanation: a separation of time-scales of the processes involved. Selection and population demographic changes drive divergence of populations during isolation. Migration and recombination during population reconnection allows a rapid – relative to divergence – genome admixture. Both impact genome polymorphism, but the latter does so more drastically and at much shorter time-scales.

The evolutionary history of an organism is a powerful resource for understanding its current evolutionary trajectory. The analytical framework developed can be applied to understanding the potential evolutionary pathway of many pathogens and other species that are characterized by periods of isolation. These events are common features of pathogens (Karlsson *et al.* 2014), but also occur in numerous other species, including humans and domesticated plants and animals (Ellstrand *et al.* 1999; Lecis *et al.* 2006; Caicedo *et al.* 2007; Green *et al.* 2010; Patterson *et al.* 2012). Reconnection following a period of isolation is increasingly recognized as an effective mechanism to trigger fast and complex adaptation (Seehausen 2002, 2004; Aguilée *et al.* 2013). Following reconnection, accumulated diversity (from previously isolated populations) is shared, and the resulting high level of genetic diversity may increase the potential for adaptation (Alcala and Vuilleumier 2014). Admixture among divergent populations may also promote complex adaptation by bringing together new functions acquired in different environments that have evolved independently but are compatible with the genome. Such processes have been observed in influenza and in HIV, for example. Indeed, recombination between strains previously isolated in their nonhuman hosts preceded the HIV epidemic in humans (Bailes *et al.* 2003; Sharp and Hahn 2010; Vuilleumier and Bonhoeffer 2015). Such events are expected to be more frequent in the future due to the increase in human mobility (Karlsson *et al.* 2014). Finally, our analytical framework could also be applied to evolutionary trajectories of other zoonotic viruses, such as Ebola virus, coronaviruses, hantaviruses, or henipaviruses.

It is important to keep in mind that viral population isolation and reconnection events can occur at a much smaller time scale within hosts. There is increasing evidence suggesting that genetic compartmentalization of viral populations occurs in different organs, tissues, or cells, and might play a critical role for virus evolution and more importantly for its control. This has been demonstrated for HIV (Schnell *et al.* 2011; Buzón *et al.* 2011) as well as human cytomegalovirus (Renzette *et al.* 2011, 2013, 2014, 2015). Appling the analytical framework detailed here could provide a powerful tool to dissect the dynamics and genetic composition of virus across different genetic compartments, in addition to those dynamics between hosts.

## Appendix

### Derivation of the Theoretical Signature of Population Reconnection on Pairwise Nucleotide Differences

#### Derivation of the distribution of pairwise coalescence times:

We here derive the distribution of within- and between-population pairwise coalescence times, $T_{w}$ and $T_{b}$, assuming a long isolation period ($T_{iso}-T_{reco}>3$) and small sequences, *i.e.*, without recombination. These coalescence times $T_{w}$ and $T_{b}$ correspond to the time to the most recent common ancestor of two lineages sampled in the same and in different populations, respectively, scaled by the number of genes per population *N*.

The coalescence of two genes under the finite island model can be described by a three states continuous time Markov chain (Notohara 1990): the two genes can be present (1) in the same population, (2) in different populations or (3) be coalesced. The transition probabilities from one state to another depend on the scaled migration rate *M* between populations and on the number of populations *d*. Time *T* is counted in units of *N* generations. The corresponding Markov chain can be summarized by the following transition rate matrix (Notohara 1990):

$$Q=\left(\begin{array}{ccc} -M-1 & M & 1\\ M/\left(d-1\right) & -M/\left(d-1\right) & 0\\ 0 & 0 & 0 \end{array}\right)$$ (A.1)

The probability that two genes were in state (1), (2) or (3) *T* generations ago (row vector $P_{T}$), given that they are in state (1), (2) or (3) at the current generation is given by $P_{T}=P_{0}e^{QT}$ ($P_{0}$ at generation 0), where $e^{QT}$ is a 3×3 matrix. The elements of matrix $e^{QT}$ are denoted $q_{i,j}\left(T\right)$ and represent the probability that a gene in state *i* at the current generation was in state *j T* generations ago (*cumulative* distribution function). Thus, the probability distribution (or probability *density* function) of within-population and between-population pairwise coalescence times are given by the derivatives of corresponding $q_{i,j}\left(T\right)$ functions: $f_{w,coal}\left(T\right)=\frac{d}{dT}q_{1,3}\left(T\right)$ and $f_{b,coal}\left(T\right)=\frac{d}{dT}q_{2,3}\left(T\right)$. Populations are considered isolated when $T_{reco}<T<T_{iso}$ and no migration occurs, with M=0 in the transition matrix **Q**. Populations exchange migrants when $T<T_{reco}$ and $T>T_{iso}$, thus M>0 in the transition matrix **Q**. Consequently, the distributions of within- and between-population pairwise coalescence times, $f_{T_{w}}\left(T\right)$ and $f_{T_{b}}\left(T\right)$, following a past isolation event, are:

$$f_{T_{w}}\left(T\right)=\left\{ \begin{array}{l} f_{w,coal}\left(T\right),\;T<T_{reco}\\ q_{1,1}\left(T_{reco}\right)e^{-\left(T-T_{reco}\right)},\;T_{reco}<T<T_{iso}\\ q_{1,1}\left(T_{reco}\right)e^{-\left(T_{iso}-T_{reco}\right)}f_{w,coal}\left(T-T_{iso}\right)\\ +q_{1,2}\left(T_{reco}\right)f_{b,coal}\left(T-T_{iso}\right),\;T_{iso}<T, \end{array}\right.$$ (A.2a)

$$f_{T_{b}}\left(T\right)=\left\{ \begin{array}{l} f_{b,coal}\left(T\right),\;T<T_{reco}\\ q_{2,1}\left(T_{reco}\right)e^{-\left(T-T_{reco}\right)},\;T_{reco}<T<T_{iso}\\ q_{2,1}\left(T_{reco}\right)e^{-\left(T_{iso}-T_{reco}\right)}f_{w,coal}\left(T-T_{iso}\right)\\ +q_{2,2}\left(T_{reco}\right)f_{b,coal}\left(T-T_{iso}\right),\;T_{iso}<T. \end{array}\right.$$ (A.2b)

These distributions have a simple interpretation. In the most recent period, the reconnection period, when $T<T_{reco}$, $f_{T_{w}}\left(T\right)$ and $f_{T_{b}}\left(T\right)$ correspond to the probability of coalescence of 2 lineages under the finite island model, $f_{w,coal}\left(T\right)$ and $f_{b,coal}\left(T\right)$. During the past isolation period, $T_{reco}<T<T_{iso}$, two lineages can only coalesce if they are in the same population after $T_{reco}$ generations of connection (probabilities $q_{1,1}\left(T_{reco}\right)$ and $q_{2,1}\left(T_{reco}\right)$ within- and between-populations, respectively). Their probability of coalescence at generation *T* in an isolated population is $e^{-\left(T-T_{reco}\right)}$. During the older connection period, $T>T_{iso}$, two genes can coalesce if they were in the same population during isolation (probabilities $q_{1,1}\left(T_{reco}\right)$ and $q_{2,1}\left(T_{reco}\right)$, within- and between-populations, respectively) and if they did not coalesce during isolation (probability $e^{-\left(T_{iso}-T_{reco}\right)}$). Their probability of coalescence at generation *T* is $f_{w,coal}\left(T-T_{iso}\right)$. Alternatively, they could have coalesced if they were in different populations during isolation (probabilities $q_{1,2}\left(T_{reco}\right)$ and $q_{2,2}\left(T_{reco}\right)$). Their probability of coalescence at generation *T* is $f_{b,coal}\left(T-T_{iso}\right)$.

Functions $f_{w,coal}\left(T\right)$, $f_{b,coal}\left(T\right)$, $q_{1,1}\left(T\right)$, $q_{1,2}\left(T\right)$, $q_{2,1}\left(T\right)$ and $q_{2,2}\left(T\right)$ all depend on the eigenvalues of matrix **Q**, $\lambda_{1}=-\frac{1}{2}\left(1+\frac{d}{d-1}M+\sqrt{\Delta }\right)$ and $\lambda_{2}=-\frac{1}{2}\left(1+\frac{d}{d-1}M-\sqrt{\Delta }\right)$, where $\Delta ={\left(d-1+dM\right)}^{2}-4\left(d-1\right)M$. We obtain:

$$\begin{array}{l} f_{w,coal}\left(T\right)=A_{w,1}\lambda_{1}e^{\lambda_{1}T}+A_{w,2}\lambda_{2}e^{\lambda_{2}T}\\ f_{b,coal}\left(T\right)=A_{b,1}\lambda_{1}e^{\lambda_{1}T}+A_{b,2}\lambda_{2}e^{\lambda_{2}T} \end{array}$$ (A.3a)

$$\begin{array}{l} q_{1,1}\left(T\right)=\left(B+A_{w,1}\right)e^{\lambda_{1}T}-\left(B-A_{w,2}\right)e^{\lambda_{2}T}\\ q_{1,2}\left(T\right)=-Be^{\lambda_{1}T}+Be^{\lambda_{2}T}\\ q_{2,1}\left(T\right)=\frac{-B}{d-1}e^{\lambda_{1}T}+\frac{B}{d-1}e^{\lambda_{2}T}\\ q_{2,2}\left(T\right)=\left(-B+A_{w,2}\right)e^{\lambda_{1}T}+\left(B+A_{w,1}\right)e^{\lambda_{2}T}. \end{array}$$ (A.3b)

Where $A_{w,1}=\frac{1+\lambda_{2}}{\lambda_{2}-\lambda_{1}}$, $A_{w,2}=\frac{-\left(1+\lambda_{1}\right)}{\lambda_{2}-\lambda_{1}}$, $A_{b,1}=\frac{\lambda_{2}}{\lambda_{2}-\lambda_{1}}$, $A_{b,2}=\frac{-\lambda_{1}}{\lambda_{2}-\lambda_{1}}$ and $B=\frac{M}{\lambda_{2}-\lambda_{1}}$.

#### Derivation of the distribution of pairwise nucleotide differences:

From equations A.2a and A.2b, we can derive the distributions of within- ($\pi_{w}$) and between-population pairwise nucleotide differences ($\pi_{b}$). Given that mutations follow a Poisson process of mean θ, we have $P\left(\pi_{w}=k/G\right)=\int_{0}^{\infty }e^{-\theta T}\frac{{\left(\theta T\right)}^{k}}{k!}f_{T_{w}}\left(T\right)dT$ and $P\left(\pi_{b}=k\right)=\int_{0}^{\infty }e^{-\theta T}\frac{{\left(\theta T\right)}^{k}}{k!}f_{T_{b}}\left(T\right)dT$, where *k* is a positive integer and *G* is the length of the sequence (under the infinite size model, we assume that *G* is much larger than the number of segregating site). We can decompose $P\left(\pi_{w}=k\right)$ into a sum of smaller integrals (respectively for $P\left(\pi_{b}=k\right)$):

$$\begin{array}{r} P\left(\pi_{w}=\frac{k}{G}\right)=\int_{0}^{T_{reco}}e^{-\theta T}\frac{{\left(\theta T\right)}^{k}}{k!}f_{T_{w}}\left(T\right)dT+\int_{T_{reco}}^{T_{iso}}e^{-\theta T}\frac{{\left(\theta T\right)}^{k}}{k!}f_{T_{w}}\left(T\right)dT\\ +\int_{T_{iso}}^{\infty }e^{-\theta T}\frac{{\left(\theta T\right)}^{k}}{k!}f_{T_{w}}\left(T\right)dT. \end{array}$$ (A.4)

Substituting the value of $f_{T_{w}}\left(T\right)$ in each term of equation A.4 by the corresponding value in equation A.2a and using the result $\int_{T_{1}}^{T_{2}}e^{-\theta T}\frac{{\left(\theta T\right)}^{k}}{k!}\lambda e^{\lambda T}dT=\frac{{\left(-\frac{\theta }{\lambda }\right)}^{k}}{{\left(1-\frac{\theta }{\lambda }\right)}^{k+1}}\left[e^{\left(\lambda -\theta \right)T_{2}}\sum_{l=0}^{k}\frac{T_{2}^{l}{\left(-\lambda +\theta \right)}^{l}}{l!}-e^{\left(\lambda -\theta \right)T_{1}}\sum_{l=0}^{k}\frac{T_{1}^{l}{\left(-\lambda +\theta \right)}^{l}}{l!}\right]$, we obtain:

$$\begin{array}{c} P\left(\pi_{w}=\frac{k}{G}\right)=\sum_{i=0}^{2}A_{w,i}\frac{{\left(-\frac{\theta }{\lambda_{i}}\right)}^{k}}{{\left(1-\frac{\theta }{\lambda_{i}}\right)}^{k+1}}\left(1-e^{\left(\lambda_{i}-\theta \right)T_{reco}}\sum_{j=0}^{k}\frac{T_{reco}^{l}{\left(-\lambda_{i}+\theta \right)}^{l}}{l!}\right)\\ +q_{1,1}\left(T_{reco}\right)e^{T_{reco}}\frac{\theta^{k}}{{\left(1+\theta \right)}^{k+1}}[e^{-\left(1+\theta \right)T_{reco}}\sum_{l=0}^{k}\frac{T_{reco}^{l}{\left(1+\theta \right)}^{l}}{l!}\\ -e^{-\left(1+\theta \right)T_{iso}}\sum_{l=0}^{k}\frac{T_{iso}^{l}{\left(1+\theta \right)}^{l}}{l!}]\\ +\sum_{i=0}^{2}\left[(\frac{{\left(-\frac{\theta }{\lambda_{i}}\right)}^{k}}{{\left(1-\frac{\theta }{\lambda_{i}}\right)}^{k+1}}e^{-\theta T_{iso}}\sum_{l=0}^{k}\frac{T_{iso}^{l}{\left(-\lambda_{i}+\theta \right)}^{l}}{l!}\right)\\ \left(q_{1,1}\left(T_{reco}\right)e^{-T_{iso}+T_{reco}}A_{w,i}+q_{1,2}\left(T_{reco}\right)A_{b,i}\right)] \end{array}$$ (A.5)

The resulting distributions of pairwise nucleotide differences (from equation A.5) are presented in Figure 1A. Further assuming that $T_{iso}-T_{reco}>3$ (thus $e^{-T_{iso}+T_{reco}}≃0$) leads to:

$$\begin{array}{c} P\left(\pi_{w}=\frac{k}{G}\right)=\sum_{i=0}^{2}\frac{A_{w,i}{\left(-\frac{\theta }{\lambda_{i}}\right)}^{k}}{{\left(1-\frac{\theta }{\lambda_{i}}\right)}^{k+1}}\left(1-e^{\left(\lambda_{i}-\theta \right)T_{reco}}\sum_{j=0}^{k}\frac{T_{reco}^{l}{\left(-\lambda_{i}+\theta \right)}^{l}}{l!}\right)\\ +q_{1,1}\left(T_{reco}\right)\frac{\theta^{k}}{{\left(1+\theta \right)}^{k+1}}e^{-\theta T_{reco}}\sum_{l=0}^{k}\frac{T_{reco}^{l}{\left(1+\theta \right)}^{l}}{l!}\\ +q_{1,2}\left(T_{reco}\right)\sum_{i=0}^{2}\frac{A_{b,i}{\left(-\frac{\theta }{\lambda_{i}}\right)}^{k}}{{\left(1-\frac{\theta }{\lambda_{i}}\right)}^{k+1}}e^{-\theta T_{iso}}\sum_{l=0}^{k}\frac{T_{iso}^{l}{\left(-\lambda_{i}+\theta \right)}^{l}}{l!}, \end{array}$$ (A.6a)

$$\begin{array}{c} P\left(\pi_{b}=\frac{k}{G}\right)=\sum_{i=0}^{2}\frac{A_{b,i}{\left(-\frac{\theta }{\lambda_{i}}\right)}^{k}}{{\left(1-\frac{\theta }{\lambda_{i}}\right)}^{k+1}}\left(1-e^{\left(\lambda_{i}-\theta \right)T_{reco}}\sum_{j=0}^{k}\frac{T_{reco}^{l}{\left(-\lambda_{i}+\theta \right)}^{l}}{l!}\right)\\ +q_{2,1}\left(T_{reco}\right)\frac{\theta^{k}}{{\left(1+\theta \right)}^{k+1}}e^{-\theta T_{reco}}\sum_{l=0}^{k}\frac{T_{reco}^{l}{\left(1+\theta \right)}^{l}}{l!}\\ +q_{2,2}\left(T_{reco}\right)\sum_{i=0}^{2}\frac{A_{b,i}{\left(-\frac{\theta }{\lambda_{i}}\right)}^{k}}{{\left(1-\frac{\theta }{\lambda_{i}}\right)}^{k+1}}e^{-\theta T_{iso}}\sum_{l=0}^{k}\frac{T_{iso}^{l}{\left(-\lambda_{i}+\theta \right)}^{l}}{l!}. \end{array}$$ (A.6b)

Terms in equation A.6a (resp. A.6b) have important interpretations in terms of coalescent events. The first term is the contribution of sequences which coalesce during the recent connection period; indeed, $\sum_{i=0}^{2}A_{w,i}\frac{{\left(-\frac{\theta }{\lambda_{i}}\right)}^{k}}{{\left(1-\frac{\theta }{\lambda_{i}}\right)}^{k+1}}$ (resp. $\sum_{i=0}^{2}A_{b,i}\frac{{\left(-\frac{\theta }{\lambda_{i}}\right)}^{k}}{{\left(1-\frac{\theta }{\lambda_{i}}\right)}^{k+1}}$) corresponds to the distribution of $\pi_{w}$ (resp. $\pi_{b}$) under the finite island model at equilibrium, while terms $\left(1-e^{\left(\lambda_{i}-\theta \right)T_{reco}}\sum_{j=0}^{k}\frac{T_{reco}^{l}{\left(-\lambda_{i}+\theta \right)}^{l}}{l!}\right)$ are due to the truncation of the distribution between $T_{reco}$ and infinity. The second term corresponds to sequences that coalesce during the isolation period. Indeed, with probability $q_{1,1}\left(T_{reco}\right)$ (resp. $q_{2,1}\left(T_{reco}\right)$), sequences did not coalesce during the reconnection period and were in the same population during isolation; $\frac{\theta^{k}}{{\left(1+\theta \right)}^{k+1}}$ correspond to the distribution of $\pi_{w}$ in an isolated population, and the term $e^{-\theta T_{reco}}\sum_{l=0}^{k}\frac{T_{reco}^{l}{\left(1+\theta \right)}^{l}}{l!}$ is due to the truncation between 0 and $T_{reco}$. Finally, the third term corresponds to sequences that coalesce during the prior connection period. With probability $q_{1,2}\left(T_{reco}\right)$ (resp. $q_{2,2}\left(T_{reco}\right)$), sequences coalesce during the prior connection period (as they did not coalesce during the reconnection period and were in different populations during isolation). Then, $\sum_{i=0}^{2}A_{b,i}\frac{{\left(-\frac{\theta }{\lambda_{i}}\right)}^{k}}{{\left(1-\frac{\theta }{\lambda_{i}}\right)}^{k+1}}$ corresponds to the distribution of $\pi_{b}$ under the finite island model at equilibrium, while terms $e^{-\theta T_{iso}}\sum_{j=0}^{k}\frac{T_{iso}^{l}{\left(-\lambda_{i}+\theta \right)}^{l}}{l!}$ are due to the truncation of the distribution between 0 and $T_{iso}$.

#### Derivation of the power to detect bimodality:

We can derive the power to detect bimodality from the distributions of $\pi_{w}$ and $\pi_{b}$ by computing the probability that, in a sample of *n* independent pairwise differences sequences, at least one is from the first mode and at least one is from the second. Given that the probability for a difference $\pi_{w}$ to be drawn from the second mode of the distribution is $q_{1,2}\left(T_{reco}\right)$, the probability that among *n* differences, at least one is from the first mode and one from the second mode is $1-q_{1,2}{\left(T_{reco}\right)}^{n}-{\left(1-q_{1,2}\left(T_{reco}\right)\right)}^{n}$. Similarly, the probability to observe a bimodal distribution of $\pi_{b}$ from a sample of size *n* is $1-q_{2,2}{\left(T_{reco}\right)}^{n}-{\left(1-q_{2,2}\left(T_{reco}\right)\right)}^{n}$. These results are used to draw Figure A in File S1. Note that with *n* sequences, we can only compute n−1 independent pairwise differences, although the total number of pairwise differences we can compute is $\frac{n\left(n-1\right)}{2}$.
